# Clinical Efficacy Evaluation and Potential Mechanism of Zhishe Tongluo Capsule in the Treatment of Cerebral Infarction by Meta-Analysis Associated with Network Pharmacology

**DOI:** 10.1155/2022/2471927

**Published:** 2022-01-10

**Authors:** Zhi Xin Geng, Feng Gao, Junjing Guo, Bingzhou Guo, Chunyu Liu, Taiwei Dong, Min Li, Yang Bai, Peifeng Wei

**Affiliations:** ^1^Shannxi University of Chinese Medicine, Xianyang 712046, China; ^2^Shaanxi Jianmin Pharmaceutical Co Ltd, Xianyang 712021, China; ^3^The Second Affiliated Hospital of Shaanxi University of Chinese Medicine, Xianyang 712000, China

## Abstract

**Objective:**

By integrating meta-analysis and network pharmacology strategy, the clinical efficacy of Zhishe Tongluo capsule in the treatment of cerebral infarction was evaluated, and the intervention mechanism was preliminary explored.

**Methods:**

Through meta-analysis, the Chinese and English literature of the randomized controlled trial (RCT) of Zhishe Tongluo capsule in the treatment of cerebral infarction was comprehensively searched. Based on the standard of Na Pai, the quantitative literature was determined and the Review Manager data were statistically analyzed.

**Results:**

A total of 10 RCTs literatures were included. These literatures included a total of 1278 subjects, of which 670 were in the treatment group and 608 were in the control group. In terms of indicators of efficiency and adverse reaction rate, the treatment group was better than the control group. There was a statistical difference (*P* < 0.05); a total of 559 chemical constituents and 2306 potential targets were obtained from the online database. Of these, 201 components, 145 targets, and 185 pathways were closely related to cerebral infarction.

**Conclusions:**

The available evidence indicates that the addition of Zhishe Tongluo capsule to the conventional treatment of Western medicine can improve the clinical efficacy of cerebral infarction and has some advantages in regulating blood lipids and hemorheology, but the overall evidence level is low, which still needs to be further supported by large-scale and multicenter RCTs; intervention of brain infarction by Zhishe Tongluo capsule is a comprehensive result of multicomponent and multi-target interactions. On the basis of the combined meta-analysis and network pharmacology in scientific attempts, it also provides a reference for the clinical evaluation of other drugs and mechanism research.

## 1. Introduction

According to the statistics, when it becomes available to approximately 55.5 million people every year worldwide without receiving timely treatment, infarct in the anterior bulb is currently a global concern [1, 2], and studies of this disease have shown that the morbidity and mortality of the disease are submaximal in Asia [3], especially in China and Eastern Europe. In addition, according to the mainland burden report, if the incidence of cerebral infarction does not change, the total number of deaths from cerebral infarction will reach more than 30% in 2035 [4, 5]. Infarct is fast and severe, and long-term care is required for the treatment, the incidence of cerebral infarction is fast and harmful, and the long-term care is required for the treatment follow-up [6], in which social development greatly imposes a large burden on socioeconomic development [7]. Cerebral infarction, also known as ischemic stroke, refers to the syndrome of ischemia and hypoxia-induced necrosis and softening of brain tissue resulting from local blood supply disorders in the brain, thereby producing symptoms of corresponding brain functional deficits, and is characterized by high mortality and disability [8–10]. In the treatment of cerebral infarction, traditional Chinese medicine (TCM) focuses on the holistic concept, syndrome differentiation, and treatment, while Western medicine focuses on local and systematic medication. The knot is important for the clinical differentiation of traditional Chinese medicine (TCM) according to the symptom, symptom, and combination of syndromes [11]. The multitarget effects of TCMs in the treatment of cerebral infarction fully play the advantages of adjusting the function of the organism, multilevel, and all-faceted treatment of patients based on syndrome differentiation, while also considering at the cellular, molecular, and genetic levels. The clinical effects of cerebral infarction patients treated with traditional Chinese medicine, Chinese patent medicine, or combination drugs are quite ideal, which can not only alleviate the acute symptoms of cerebral infarction, but also play a role in blood lipid indicators, neurological deficits, and later recovery of daily living ability, so we should make an active promotion in the clinic. Some studies have shown that Chinese patent medicine Zhishe Tongluo capsule (ZTC), produced by Jianmin Pharmaceutical in Shaanxi, has an obvious ameliorative effect on the treatment of cerebral infarction and multiple indications during the recovery period of patients, which is composed of Radix Astragali, Radix Ginseng, Rhizoma Gastrodiae, Radix Salviae Miltiorrhizae, Radix Puerariae, Rhizoma Chuanxiong, Radix gladioli, depression, Radix Hirudinea, borneol, and Radix Aconiti Lateralis, and has the efficacy of invigorating Qi, invigorating blood, and relieving wind and collaterals. It is mainly used for Qi deficiency and blood stasis syndrome in the recovery phase of meridian (mild-to-moderate cerebral infarction) in stroke disease. To further demonstrate the advantages of this medicine in the treatment of cerebral infarction, in this study, the means of using meta-analysis and network pharmacology were analyzed in more depth, which provided strong evidence-based support for better promotion and application of ZTC in clinical.

Based on the above research status, this study is first based on meta-analysis, with the conventional Western medicine diagnosis and treatment as the control group and ZTC combined with the conventional treatment as the observation group, to evaluate the therapeutic effect of the two groups, a comprehensive and comprehensive analysis of the effective rate of patients with cerebral infarction, the incidence of adverse reactions, blood lipid levels, and other outcome indicators, which provide evidence-based support for clinical application. In addition, on the basis of meta-analysis, further application of network pharmacology method and the potential mechanism of ZTC in the treatment of cerebral infarction and other cardiovascular and cerebrovascular diseases were preliminary explored to provide a reference for subsequent molecular biology research.

## 2. Method

### 2.1. Meta-Analysis

#### 2.1.1. Inclusion Criteria

The inclusion criteria were as follows: (1) patients were diagnosed with cerebral infarction, ischemic stroke, etc. The main clinical manifestations were headache, vertigo, nausea and vomiting, motor and/or sensory aphasia, and even coma. In severe cases, stare at the side of the lesion, central facial paralysis, and tongue paralysis. The course of the disease is more than 3 months. (2) Research scheme: the treatment group was given ZTC, and the control group was given conventional treatment. (3) The main efficacy indicators include treatment efficiency, incidence of adverse reactions, blood lipid TG level, blood lipid LDL-C level, blood lipid HDL-C level, and inefficiency. (4) Research type is RCT. (5) Literature language is limited to Chinese and English.

#### 2.1.2. Exclusion Criteria

Exclusion criteria are as follows: (1) participants suffered from other serious diseases, (2) patients with allergic reactions to the drugs used, (3) repeated published literature, (4) nonrandomized controlled trials, and (5) literature studies that lack data on the above indicators cannot be obtained.

### 2.2. Literature Retrieval Strategy

Two researchers searched the English and Chinese literature on ZTC in the treatment of cerebral infarction and ischemic stroke. Seven databases were searched, including CNKI, CBM, VIP, WanFang, PubMed, EMBASE, and Cochrane. Chinese search terms are as follows: ZTC, cerebral infarction, ischemic stroke, etc. The keywords in English were ‘ZTC' AND ‘Cerebral infarction' OR ‘ISCHEMIC stroke'.

### 2.3. Literature Screening and Data Extraction

Combined with the relevant inclusion and exclusion criteria of the literature, the two researchers independently screened the literature and extracted and sorted out the relevant data. If there were differences, the third researcher could help to discuss and solve them. Baseline information extracted included year of first author and publication, random method, number of cases, age, gender, specific interventions and treatment processes, and outcome indicators.

### 2.4. Bias Risk Assessment

The two researchers assessed the bias risk included in the literature according to the bias risk assessment tool recommended by the Cochrane Handbook of Systematic Reviewers. The methodological criteria for evaluation are as follows: (1) generation of random sequences, (2) distribution hiding, (3) blind method, (4) result data integrity, (5) reporting bias, and (6) other prejudices. We assessed the bias risk of each RCT and divided it into “low risk,” “high risk,” or “unclear risk.” The above operations were independently completed by two researchers. If necessary, a third researcher could participate in the negotiations and eventually reach consensus.

### 2.5. Statistical Analysis

Meta-analysis was performed using Cochrane Reviewer Manager 5.2 Software. Binary variables use odds ratio (OR) statistical effect, continuous variables use mean difference (MD) statistical effect, and effect size provides 95% confidence interval (CI). The chi-square test was used to analyze the heterogeneity among the included research results, and the *P* value and *I*^2^ were used to evaluate whether there was heterogeneity. If the heterogeneity among the studies was low (*P* ≥ 0.10 and *I*^2^ ≤ 50%), the fixed-effects model was used. If the heterogeneity is high (*P* < 0.10 or *I*^2^ ≥ 50%), the random-effects model is used for analysis. In addition, if there is significant heterogeneity between each research result, subgroup analysis or sensitivity analysis can be used to detect the causes of heterogeneity, or only descriptive analysis can be used.

### 2.6. Network Pharmacology Method

#### 2.6.1. Establishment of Chemical Constituents Library of ZTC

Based on Bioinformatics Analysis Tool for Molecular Mechanism of Traditional Chinese Medicine (BATMAN-T CM), (http://bionet.ncpsb.org/batman-tcm/), Traditional Chinese Medicine Systems Pharmacology (TCMSP) [12], (http://lsp.nwu.edu.cn/tcmsp.php), OMIM [13] (https://omim.org/), and Traditional Chinese Medicine Integrated Database (TCMID) (http://183.129.215.33/tcmid/), the chemical constituents of ZTC were collected and the chemical constituents library was constructed.

#### 2.6.2. Construction of the Database of Chemical Constituent Targets

The construction of “chemical composition target” comes from two aspects: (1) prediction based on online data: STITCH (https://stitch.embl.de/), DRAR-CPI (https://cpi.bio-x.cn/drar/), and Swiss TargetPrediction (https://www.swisstargetprediction.ch/) were used to predict the target of chemical composition in ZTC and (2) prediction based on traditional Chinese medicine database: TCMSP, BANTAM AN-T CM, and TCMID, and other databases were used to screen and integrate the action targets of each chemical component, and UniProt data [14] (https://www.uniprot.org/) were used to correct the target as the standard gene name of the official source.

### 2.7. Construction of “Cerebral Infarction Target” Database

“Cerebral infarction” is used as the search term to search OMIM database (https://omim.org/), GeneCards database (https://www.genecards.org/), and DisGeNET database (https://www. disgenet.org/). When searching the OMIM database, “Gene Map” is clicked after opening the Web page and the search term is entered in the search box to get the disease genes in the database; when searching the GeneCards database, the keyword search box is entered after opening the Web page and you can get the disease genes in the database by searching the search term; and when searching the DisGeNET database, “search” is clicked after opening the Web page, “diseases” is selected, and the search term is entered in the search box beneath to get the information in the database. Finally, the genes obtained in the above-mentioned database are summarized, and duplicate genes are deleted.

#### 2.7.1. Construction of Protein Interaction Network between Targets and Screening of Core Targets

In network pharmacology studies, string is generally applied to construct a protein albumin interaction network between target proteins using the white database of structural target proteins. As a new drug research method, PPI network can be used to clarify the relationship between predicted targets and other proteins. The STRING database (https://string-db.org) is a database for searching protein interactions. It provides information about protein predictions and interactions. The STRING database is used to construct the PPI network of ZTC for the treatment of cerebral infarction, and it is visualized with Cytoscape Software. After deduplication, the intersection target processed by the Venn graph is imported into the STRING database, and the organism is set to “Homo sapiens” to search, the highest confidence level of the gene data (score ≥ 0.9) is taken, the processed data are imported into Cytoscape 3.7.1 Software [15], the software's built-in Network Analysis plug-in is used for network topology analysis, and protein-protein interaction network and screen core targets are constructed.

#### 2.7.2. GO Enrichment Analysis and KEGG Pathway Enrichment Analysis of Common Targets of Drugs and Diseases

First, the potential target of 1.4 was converted into Entrez ID using *R* language software [16]. Then, clusterProfiler and Bioconductor packages for *R* language software were installed. The results of KEGG enrichment analysis were obtained by running *R* language and displayed in the form of strip diagram, bubble diagram, and pathway diagram. The significance of enrichment function and pathway was judged according to *P* value to explore the possible mechanism of ZTC in the treatment of cerebral infarction.

### 2.8. Molecular Docking

tThe target structure is downloaded from the RCSB PDB database (http://www.rcsb.org), and the PyMOL software is used to remove the crystal water and other small molecules of the target structure, it is saved in the pdb format, and then the structure file is placed in AutoDockTools 1.5.6 program, the atomic charge is added, and it is saved in pdbqt format after adding hydrogen atom. The mol2 format structure file of the key active ingredient of ZTC is put into the AutoDockTools program, the atomic charge is added, and it is saved in pdbqt format as the docking ligand. After using AutoDock Vina software to perform operations such as water removal, hydrogenation, and selection as a ligand, the molecular docking was simulated and visualized in PyMOL. To determine the binding affinity of the target protein and potential biologically active ingredients [17], the binding affinity between these biologically active ingredients and the target protein is used as an evaluation criterion. The smaller the binding affinity, the stronger the binding ability.

## 3. Results

### 3.1. Meta-Analysis Results

#### 3.1.1. Literature Screening Process and Results

**Figure 1:**
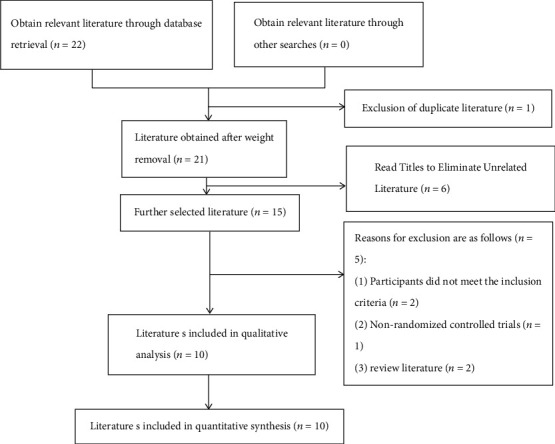
Literature screening process and results.

A total of 22 related literature studies were obtained by preliminary search. After using NoteExpress to eliminate duplicate literature, 21 articles were obtained. After reading the titles and abstracts, 6 unrelated articles were eliminated, and then, the full text was searched and read to further exclude the literature that did not meet the inclusion criteria, nonrandomized controlled trials, and literature review, and finally, 10 RCTs were included. All RCTs were conducted in China and published in Chinese. The flowchart and literature screening results are shown in Figure 1.

#### 3.1.2. Baseline Characteristics Included in the Study

A total of 10 RCTs were included, including 1278 patients, including 670 cases in the treatment group and 608 cases in the control group. The treatment time of all patients was mostly 4 weeks, and the study showed that the treatment group and the control group were comparable in terms of gender, age, condition, and course of disease. In the control group, five studies referred to the conventional treatment and five studies increased aspirin, atorvastatin, and other drugs. On the basis of the treatment measures of the control group, the treatment group was treated with ZTC. The outcome indicators include treatment efficiency, blood lipid TG level, blood lipid HDL-C level, adverse reactions, and inefficiency.  Table 1 lists the detailed basic characteristics of the included studies.

#### 3.1.3. Bias Risk Assessment of Included Studies

**Figure 2:**
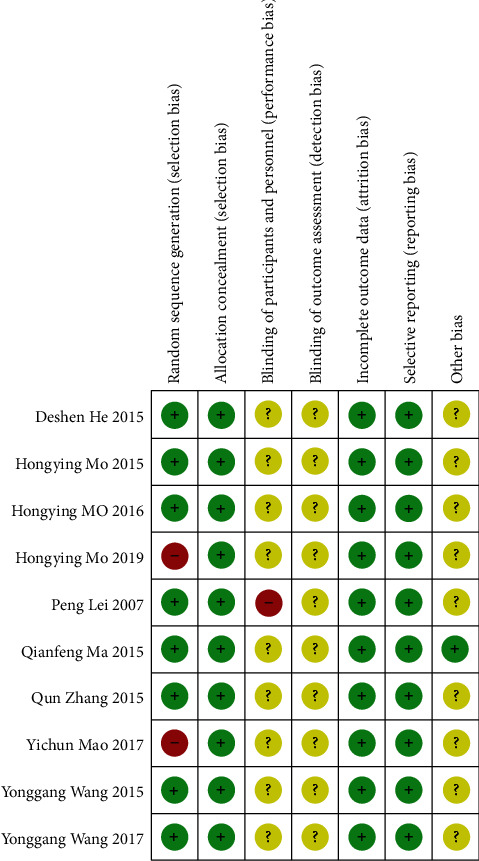
Bias risk assessment.

According to the bias risk assessment method recommended by Cochrane, the four included studies used the random number table method to group the patients and evaluated them as “low risk.” One study used the computer random grouping method to group the patients and evaluated them as “low risk.” One study grouped the patients by drawing lots and evaluated them as “low risk.” One study was described as grouping the patients according to the odd and even numbers of the admission date and evaluated them as “high risk.” The remaining studies did not describe specific random methods, assessed as “unclear risk.” All studies did not describe the hidden allocation, did not mention the use of blind method, and did not mention the subjects who exited in the middle. Other potential sources of bias are still unclear. See Table 1 for details. The summary of bias risks included in the study is shown in Figure 2.

#### 3.1.4. Comprehensive Analysis

**Figure 3:**
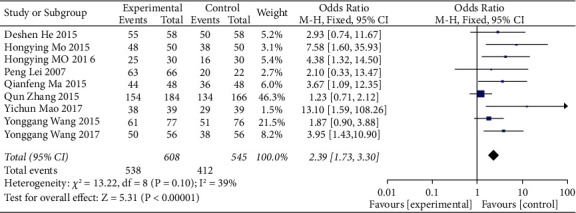
Meta-analysis of the effective rate of conventional treatment combined with the ZTC group and the conventional treatment group.

A total of 9 studies on the effective rate of treatment were used as an indicator of efficacy evaluation. The effective rate of treatment is the statistical index of other cases except invalid cases recorded in the reference literature after drug treatment, including recovery, marked effect, and effectiveness. The effective rate of treatment is the proportion of the total number of people taking the medicine, including healing, marked effect, and effectiveness, in other words the proportion of the number of people who have improved symptoms, signs, or laboratory tests after taking the medicine including 1875 patients, and the heterogeneity between the study groups was low (*P* = 0.10, I^2^ = 39%). Meta-analysis showed (Figure 3) that the effective rate of the treatment group was better than that of the simple treatment group (OR = 2.39, 95% CI = (1.73, 3.30), *P* < 0.00001), and the difference was statistically significant.

**Figure 4:**
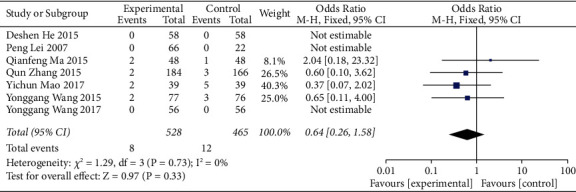
Meta-analysis of the incidence of adverse reactions between the conventional treatments combined with the ZTC group and the conventional treatment group.

*(1) Safety*. There were 7 references to the incidence of adverse reactions, and heterogeneity analysis showed no heterogeneity between studies (*P*=0.73, *I*^2^=0%). The results of meta-analysis using the fixed-effects model showed that there was no significant difference between the treatment group and the control group (OR = 0.64, 95% CI = (0.26, 1.58), *P*=0.33), as shown in Figure 4.

**Figure 5:**
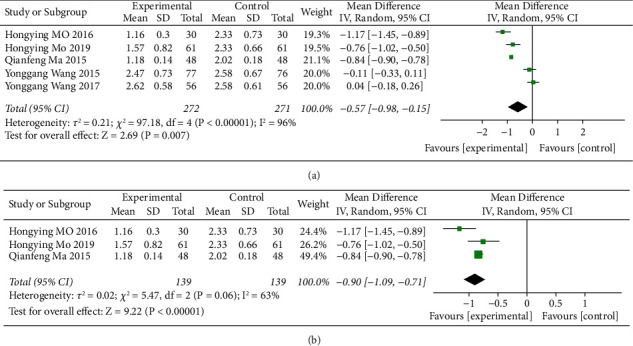
(a) Meta-analysis of the comparison of TG between the conventional treatments combined with the ZTC group and the conventional treatment group. (b) Meta-analysis of TG heterogeneity in reducing blood lipids.

*(2) Blood Lipid Level*. A total of five literature studies referred to the level of blood lipid TG, and there was a high degree of heterogeneity between the study groups (*P* < 0.00001, *I*^2^=96%). The random-effects model was used. The results showed that the addition of ZTC and the simple treatment group had statistical significance in the level of blood lipid TG, as shown in Figure 5(a). The sensitivity analysis was carried out by “single-elimination method” to explore the source of heterogeneity. It was found that the heterogeneity was mainly from literature 3 and literature 4. After eliminating the literature, the heterogeneity between the literature studies was significantly reduced (*I*^*2*^ = 63%). The random-effects model was used to analyze the results. The results show that there is a significant difference between the ZTC group and the drug-only group, which is statistically significant (MD = −0.90, 95% CI = (−1.09, −0.71), *P* < 0.00001), as shown in Figure 5(b).

**Figure 6:**
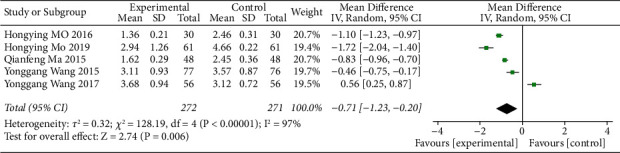
Meta-analysis of the comparison of LDL-C between the conventional treatments combined with the ZTC group and the conventional treatment group.

A total of 5 literature studies recorded the LDL-C value of blood lipids, and there was a high heterogeneity among the studies (*I*^*2*^ = 97%). The random-effects model analysis showed that there was a significant difference in LDL-C between the ZTC group and the drug-only group, which was statistically significant (MD = −0.71, 95% CI = (−1.23, −0.20), *P* < 0.00001), as shown in Figure 6.

**Figure 7:**
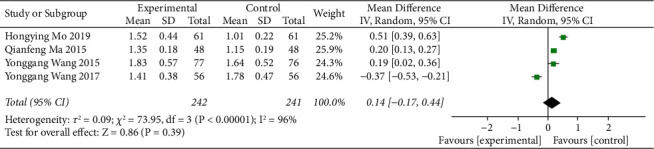
Meta-analysis of HDL-C comparison between conventional treatments combined with the ZTC group and the conventional treatment group.

HDL-C values of blood lipids were recorded in 4 articles, and there was high heterogeneity among studies (*I*^*2*^ = 97%). The results of random-effects model analysis showed that there was no significant difference in HDL-C levels of blood lipids between the ZTC group and the simple drug group (MD = 0.14, 95% CI = (−0.17, 0.44), *P*=0.39), as shown in Figure 7.

### 3.2. Results of Network Pharmacology

#### 3.2.1. Active Ingredient Screening and Target Prediction Results of ZTC

According to the conditions of OB ≥ 30% and DL ≥ 0.18 in the TCMSP database, the main active components of ZTC were screened out as *Astragalus* membranaceus 20, Ginseng 22, Gastrodia elata 11, Salvia miltiorrhiza 65, safflower 22, Pueraria lobata 4, Ligusticum chuanxiong 7, Acorus calamus 4, Curcuma 15, Hirudo 52, borneol 3, and Snake tip 7. The corresponding targets were retrieved, and the repetitive target information was screened out. Swiss TargetPrediction database was used to supplement the components without target information in TCMSP, and 424 effective target information components were obtained.

#### 3.2.2. Determination of Intersection Target between ZTC and Cerebral Infarction

**Figure 8:**
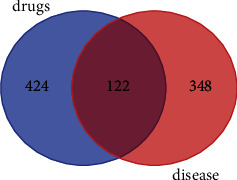
Venn diagram of drugs and disease targets.

The Venn diagram was used to collect 424 effective components of ZTC and 348 disease targets, and 122 intersection targets were obtained. See Figure 8. According to the analysis of network topology, the top three active components of degree were quercetin, HDC (3R)-3-(2-hydroxy-3,4-dimethoxyphenyl) chroman-7-o, and Sugio.

#### 3.2.3. Common Target Protein PPI Network Construction

**Figure 9:**
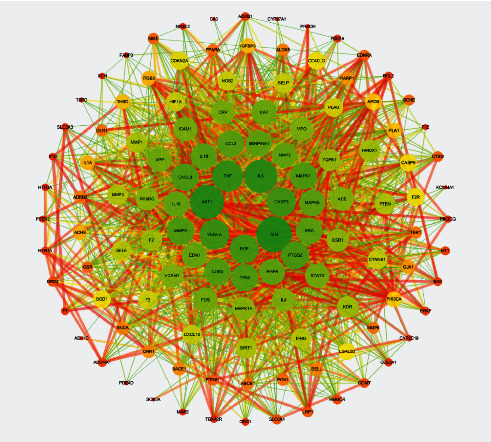
PPI network of disease targets.

**Figure 10:**
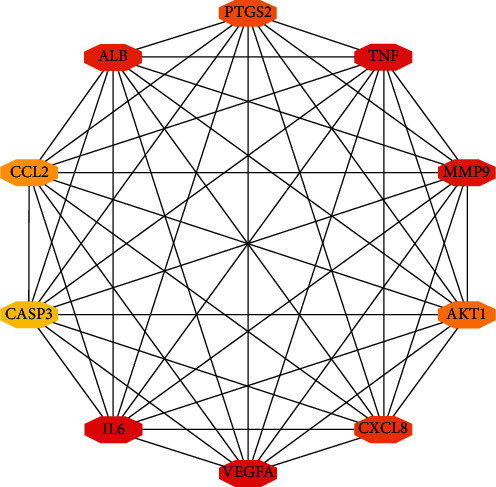
Protein map of core target.

The intersection targets of 122 mapped cerebral infarction-related diseases were analyzed through the STRING database, and the data were imported into Cytoscape 3.7.1 Software to construct the PPI network for predicting the target of ZTC in the treatment of cerebral infarction. See Figure 9. The greener the color in the figure is, the larger the area is, and the closer to the center is, the greater the degree of the target is, and the more important is it is in the network. The network has 122 nodes and 2201 edges. The network topology is analyzed with the Network Analysis plug-in Software. The average BC is 0.006, the average CC is 0.58, and the average degree is 36.08. CytoHubba plug-in is used to take the top 10° values as the core target of the network; see Figure 10 and Table 2.

#### 3.2.4. Enrichment Analysis Results and Visualization

**Figure 11:**
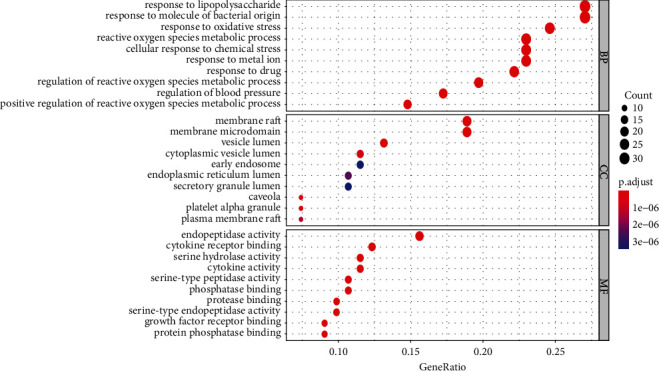
GO functional enrichment analysis bubble diagram of potential targets.

*(1) GO Enrichment Analysis*. GO function enrichment analysis is full-named “Gene Ontology.” After *R* language software processing, it shows that intersection targets enrich biological functions. The top 20 GO functional enrichment is shown in Figure 11. Round point color represents the significance of *P* value functional enrichment. The color is more red, the *P* value is smaller, and the significance is stronger. Round point size represents the number of targets involved in the function. The larger the round point, the more targets involved in the function. Therefore, ZTC in the treatment of cerebral infarction is mainly related to enzyme binding, protein binding, cell differentiation, fatty acid oxidation, and Ras protein signal transduction. The main cell components (CCs) involved in the efficacy were nuclear membrane, postsynaptic membrane, cytoplasm, and other cells.

**Figure 12:**
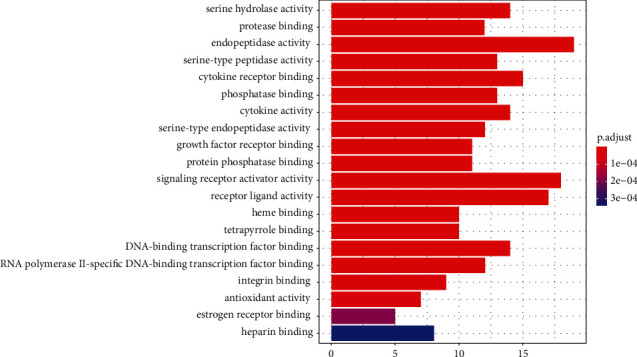
KEGG pathway enrichment analysis.

*(2) Enrichment Analysis of KEGG Pathway*. The obtained 122 intersection targets were subjected to KEGG pathway enrichment analysis using *R* language program, and 113 pathways were obtained after treatment. The top 20 pathways were plotted as a bar graph, as shown in Figure 12. The abscissa represented the number of targets involved in the pathway, and the color represented the *P* value. The more red the color was, the smaller the *P* value was and the higher the significance was after analysis, and 122 intersection targets were mainly distributed in multiple signaling pathways, such as serine hydrolase activity, protease binding, endopeptidase activity, and serine peptide activity, indicating that the main active components of ZTC could treat cerebral infarction through multiple signaling pathways, and there was a complex interaction between pathways. Multicomponent, multi-target, and mutual regulation were the possible mechanisms for the treatment of cerebral infarction.

### 3.3. Molecular Docking Verification Results

**Figure 13:**
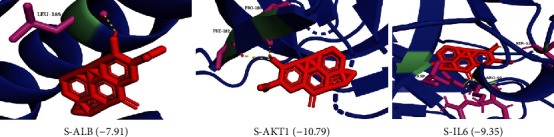
Molecular docking results.

According to the active components of “drug-component-target” network, the top three compounds with degree value were selected. The first three target proteins of the 10 key targets obtained after PPI network topology analysis were subjected to molecular docking. The smaller the free energy of molecular docking binding was, the greater the affinity between the receptor and the ligand was. The specific information is shown in Table 3. The compounds with the strongest binding to each protein were visualized by PyMOL software, as shown in Figure 13. The results showed that Sugio was the best combination with ALB, AKT1, and IL-6.

## 4. Discussion

Cerebral infarction is a serious neurological disease, and Chinese medicine belongs to the category of “stroke.” Cerebral infarction, also known as ischemic stroke or cerebral embolism, is one of the commonest cerebrovascular diseases [28]. The prevalence of cerebral infarction is high, accounting for 75%–80%. Over the last decade, China has witnessed a dramatic increase in the incidence of cerebral infarction, which is a consequence of lifestyle changing, and begins to affect the younger population [29]. ZTC is a commonly used medicine for the treatment of cerebral infarction in Chinese medicine. It is mainly composed of astragalus, ginseng, Gastrodia elata, salvia, safflower, Pueraria lobata, chuanxiong, and other traditional Chinese medicines. The combination of these drugs has the functions of promoting qi, promoting blood circulation, and dispersing wind.

Studies have shown that blood lipid levels are closely related to the occurrence of cerebral infarction. Abnormal blood lipid metabolism is also a key factor triggering cerebrovascular diseases, and the measurement of blood lipid levels is the main way to clinically monitor human lipid metabolism, which is also a major indicator for the prevention and treatment of cerebrovascular diseases [30]. Blood lipid levels include four types, TG, TC, HDL-C, and LDL-C. The levels have been considered important risk factors for ischemic cerebrovascular disease, and changes in TG levels are often accompanied by changes in LDL-C and HDL-C levels [31, 32]. Secondly, studies have demonstrated that the elevated TC levels can cause arterial stenosis and atherosclerosis [33]. The formation of atherosclerotic plaque will then lead to cerebral infarction [34]. The HDL Intervention Study for American Veterans is a clinical trial evaluating subjects with low HDL but normal total cholesterol levels. The results found that gemfibrozil can reduce the incidence of myocardial infarction and cerebral infarction, and this clinical benefit is partly attributable to HDL changes [35]. The Israeli ischemic heart disease study believes that low HDL and high TC/HDL are an independent risk factor for fatal stroke. When HDL decreases by 0.26 mmol/L, the risk of fatal stroke increases by 17% and TC/HDL per liter. The risk of fatal ischemic stroke is increased by 5% points by 18% [36]. Zhang et al. conducted a meta-analysis of 26 randomized trials (including 10 subjects) that strengthened the reduction in LDL-C and showed that for every 1 mmol/L reduction in LDL-C, heart attack, revascularization, and ischemic stroke have been reduced by more than one-fifth [37]. Therefore, we believe that blood lipid level is a vital indicator for the treatment of cerebral infarction, and we need to pay attention to it to further improve the clinical progress of the treatment of cerebral infarction.

In this study, 10 articles were finally included. Meta-analysis showed that the ZTC treatment group was significantly better than the control group in terms of effective treatment rate and TG and LDL-C, and the difference was statistically significant, and there was no statistically significant difference in HDL-C and safe. Therefore, according to the research results, it can be considered that ZTC has certain advantages in the treatment of cerebral infarction and ischemic stroke, and can improve the clinical efficacy. However, due to the small sample size, many confounding factors, and low quality of reference data, it needs to be confirmed by further large-sample, high-quality, multicenter studies.

Using network pharmacology technology to explore the potential mechanism of ZTC in the treatment of cerebral infarction, the active ingredients of ZTC and potential targets for the treatment of cerebral infarction are screened out, and a “drug-component-target,” “Dot network,” “PPI action network,” and “drug core target network” are constructed, of which ALB, CCL2, AKT1, etc., are the key core targets. The potential targets of ZTC in the treatment of cerebral infarction are enriched in signaling pathways such as serine hydrolase activity and protease binding. The potential medicinal ingredients of ZTC mainly act on core targets such as ALB, AKT1, and IL-6, and then regulate related molecular pathways to achieve the effect of treating or delaying cerebral infarction. In addition, through network topology analysis, the top three pharmaceutical ingredients with degree values are quercetin, HDC (3R)-3-(2-hydroxy-3,4-dimethoxyphenyl) chroman-7-o, Sugio, and PPI. The top three targets ALB, AKT1, and IL-6 are molecularly docked, inferring that Sugio has played a key role in the treatment. Quercetin is a natural flavonoid compound widely found in plants. It has anti-inflammatory, antioxidant, anticancer, and other pharmacological effects [38]. It can treat cerebral infarction by inhibiting and improving insulin resistance and regulating liver lipid metabolism [39]. Quercetin can regulate most of the targets, has anti-inflammatory, improves coagulation status and liver damage, etc. Chen Zhenhua et al. found that quercetin has antioxidant, anti-inflammatory, and antitumor effects and can protect the cardiomyocytes of hypoxia, widely used in the treatment of cardiovascular diseases [40]. Therefore, it may be the main compound of ZTC to exert therapeutic effects.

Although the common intervention measures were strictly controlled in this study, namely the combination of ZTC and conventional treatment, the treatment course and dose of each study were still different, so the terminal outcome effect was still different. Therefore, this study still needs more refined design, more samples, strict operation, large-scale, multicenter linkage randomized controlled trials to support. Therefore, it is suggested that clinical trials should focus on improving the quality of methodology, clearly describe the specific details of random allocation, allocation concealment, and blinding objects, standardize clinical reports, and provide better evidence-based medicine for subsequent clinical studies.

There are certain limitations in this study. (1) ZTC is a traditional Chinese medicine compound. The studies included are all domestic studies, and there are few studies abroad. Therefore, to some extent, it will also affect the conclusions of meta-analysis. (2) There are fewer studies included, resulting in a smaller sample size. Some studies include fewer patients and observations, resulting in inaccurate results [41, 42], and the lack of large-scale RCTs. (3) There may be some differences in the efficacy of the included studies with different treatment courses and doses. (4) The study included less description of adverse reactions and was unable to comprehensively evaluate the impact of interventions. (5) Network pharmacology studies ignored the influence of component content on the experimental results, and the relationship between content and efficacy should be considered in subsequent studies. (6) Network pharmacology is a research technology based on molecular network data and computer simulation analysis, so it needs to be confirmed by in vitro and in vivo experiments.

## 5. Conclusions

This study shows that ZTC has significant clinical efficacy in the treatment of cerebral infarction through the integration of meta-analysis and network pharmacology research technology. The addition of ZTC to the conventional treatment of Western medicine can improve the clinical efficacy of cerebral infarction, and it has certain advantages in lipid-lowering and neurological deficit scores, but because the quality of the included RCTs is generally low, it is still necessary to strictly follow the CONSORT standard [43] to design more large-sample, multicenter, high-quality RCTs for further verification.

## Figures and Tables

**Table 1:** Basic characteristics of included studies.

Eligible studies	Age	Sample size (T/C)	Intervention measures		Course/year	Outcome index
			T	C		
Hongying MO 2015 [18]	T: 73.32 ± 7.94 C: 71.44 ± 8.23	100 (50/50)	Conventional therapy + ZTC	Conventional therapy	Nil	①
Hongying MO 2016 [19]	T: 52.78 ± 2.56 C: 53.69 ± 3.23	60 (30/30)	Conventional therapy + atorvastatin + ZTC	Conventional therapy + atorvastatin	T: 7.89 ± 1.80 C: 7.52 ± 1.63	①③
Hongying MO 2019 [20]	—	122 (61/61)	Conventional therapy + ZTC	Conventional therapy	Nil	③⑧
Deshen He 2015 [21]	T: 45–79 C: 47–78	116 (58/58)	Conventional therapy + aspirin, atorvastatin calcium, citicoline + ZTC	Conventional therapy + aspirin, atorvastatin calcium, citicoline	T: 0.03–0.58 C: 0.03–0.58	①②
Qun Zhang 2015 [22]	T: 41–80 C: 43–78	350 (184/166)	Conventional therapy + ZTC	Conventional therapy + Xiaoshuan Tongluo tablets	Nil	①②④⑤
Yi Chun Mao 2017 [23]	T: 61.4 ± 8.7 C: 65.2 ± 8.8	78 (39/39)	Conventional therapy + ZTC	Conventional therapy	T: 0.03–0.25 C: 0.04–0.42	①②
Yonggang Wang 2015 [24]	T: 62.72 ± 12.41 C: 63.33 ± 11.71	156 (78/78)	Conventional therapy + three-stage rehabilitation of stroke + ZTC	Conventional therapy + three-stage rehabilitation of stroke	T: 0.15 ± 0.1 C: 0.14 ± 0.08	①②③⑤⑥
Yonggang Wang 2017 [25]	T: 74.7 ± 9.2 C: 73.1 ± 9.6	112 (56/56)	Conventional therapy + three-stage rehabilitation of stroke + ZTC	Conventional therapy + three-stage rehabilitation of stroke	T: 8.9 ± 3.4 C: 9.3 ± 3.7	②③⑤⑦
Peng Lei 2007 [26]	T: 60.88 ± 9.03 C: 67.18 ± 35.69	88 (66/22)	Conventional therapy + ZTC	Conventional therapy + Naoan capsule	T: 1.2 ± 0.6 C: 1.3 ± 0.69	①②④⑤
Qianfeng Ma 2015 [27]	T: 61.2 ± 8.9 C: 63.4 ± 8.2	96 (48/48)	Conventional therapy + atorvastatin + ZTC	Conventional therapy + atorvastatin	T: 5.6 ± 1.8 C: 6.7 ± 1.9	①②③

*Note.*: *T*—test group; C—control group. (1) Effective rate, (2) incidence of adverse reactions, (3) blood lipid level, (4) neurological deficit score, (5) TCM syndrome score, (6) modified Rankin scale score, and (7) hemorheology.

**Table 2:** Core targets and network topology parameters (top 10).

Gene	Degree	Betweenness centrality	Closeness centrality
ALB	98	0.08414861	0.84027778
AKT1	93	0.04878968	0.81208054
IL-6	90	0.03732065	0.79605263
TNF	83	0.02297471	0.76100629
VEGFA	78	0.01594422	0.73780488
CASP3	76	0.02091227	0.72891566
EGF	75	0.01998616	0.7245509
PTGS2	71	0.01521421	0.70760234
CXCL8	70	0.01248469	0.69942197
MMP9	70	0.00843371	0.70348837

**Table 3:** Molecular docking binding energy of active component and target protein.

Component	Chemical structural formula	CAS	Degree	Binding energy (kcal/mol)		
				ALB	AKT1	IL-6
Quercetin	C_15_H_10_O_7_	117–39–5	610	−4.7	−8.17	−6.85
HDC	C_18_H_17_NO_3_	30426–61–0	102	−5.25	−6.02	−5.27
Sugio	C_30_H_52_O	64997–52–0	101	−7.91	−10.79	−9.35

## Data Availability

The data used to support the findings of this study are included within the article.
